# Psychological Distress Among Primary Care Professionals in Singapore: Repeated-Measures Analyses of Two Independent Observation Windows (HADS and GHQ-12)

**DOI:** 10.3390/healthcare14182947

**Published:** 2026-09-10

**Authors:** Sky Wei Chee Koh, Yixuan Chuah, Si Hui Low, Alicia Ying Ying Boo, Thomas Soo, Yii Jen Lew, Howard Bauchner, Lynette Mei Lim Goh

**Affiliations:** 1 National University Polyclinics, National University Health System, Singapore 119228, Singapore; 2 Yong Loo Lin School of Medicine, National University of Singapore, Singapore 117597, Singapore; 3 School of Physical and Mathematical Sciences, Nanyang Technological University, Singapore 639798, Singapore; 4 Department of Health & Wellness, Ng Teng Fong General Hospital, National University Health System, Singapore 609606, Singapore; 5 Avedisian School of Medicine, Boston University Chobanian, Boston, MA 02118, USA

**Keywords:** primary care, healthcare professionals, psychological distress, mental health, Singapore

## Abstract

***Background:*** Healthcare professionals in Singapore’s primary care face sustained workload pressures and increasing complexity. Grounded in the Job Demands–Resources model, this study identifies demographic, clinical, and lifestyle factors associated with psychological distress among staff across two independent windows spanning the pre- and post-pandemic periods. ***Methods:*** The study was conducted among staff in National University Polyclinics (NUP) Singapore. De-identified health screening data included demographics, occupation, clinical measures, medical history, and lifestyle behaviours. Primary outcome was psychological distress, assessed across 2 distinct observation windows: the Hospital Anxiety and Depression Scale (HADS) from 2018–2022 (abnormal ≥ 8) and the General Health Questionnaire-12 (GHQ-12) from 2023–2025 (abnormal ≥ 2). Multivariate mixed-effects logistic regression identified factors associated with psychological distress while accounting for within-person clustering. ***Results:*** We analysed 3307 screening visits from 1260 staff. Within the HADS observation window (2018–2022), abnormal scores were significantly higher in 2022 compared with 2018 (OR 2.28, 95% CI 1.64–3.17). Within the GHQ-12 observation window (2023–2025), a non-significant decline in abnormal scores was observed. Older age (2018–2022: OR 0.967, 95% CI 0.948–0.987; 2023–2025: OR 0.955, 95% CI 0.928–0.983) and regular exercise (2018–2022: OR 0.724, 95% CI 0.529–0.991; 2023–2025: OR 0.357, 95% CI 0.231–0.551) were consistently associated with lower odds of distress. Medical staff had significantly lower odds of distress compared to ancillary staff in 2018–2022 (OR 0.391, 95% CI 0.207–0.739). Regular alcohol use was associated with higher distress in 2018–2022 (OR 1.56, 95% CI 1.05–2.31), while active smoking (OR 5.79, 95% CI 1.75–19.10) and family history of chronic disease (OR 2.22, 95% CI 1.28–3.87) were significant in 2023–2025. ***Conclusions:*** Older age and regular exercise were consistently associated with lower odds of psychological distress across both windows, while ancillary staff and alcohol use (HADS window), and smoking and family history of chronic disease (GHQ-12 window) were period-specific determinants. These findings support the implementation of proactive, targeted wellness initiatives alongside the routine mental health surveillance among primary care staff.

## 1. Introduction

Healthcare professionals are integral to a sustainable healthcare workforce and the delivery of high-quality patient care. In primary care settings, they contend with persistent pressures that can be understood through the Job Demands–Resources (JD-R) model [1]. According to this framework, occupational strain results from an imbalance between job demands and job resources, which are the paramount factors in achieving work goals, or reducing the psychological and physiological costs of these demands [2,3].

In the primary care context, demands include rising patient complexity, potential pandemic threats, care fragmentation, and escalating healthcare costs [4,5,6,7]. When these demands are high and resources are insufficient, the risk of health impairment processes increases tremendously. While organizational climate and institutional recognition act as critical buffers, individual-level factors may worsen resilience during tough times. Within a broad JD-R interpretive lens, pre-existing clinical conditions may function alongside occupational demands, while healthy lifestyle behaviours may serve a resource-like, buffering role.

Despite growing recognition of staff wellbeing, evidence on changes and patterns in mental health among healthcare professionals across repeated measurement periods remains limited, particularly in Singapore. Most local studies have been cross-sectional, concentrated during the COVID-19 period, capturing episodic snapshots rather than the patterns across repeated screening periods needed to understand workforce sustainability [8,9,10,11,12,13,14]. Therefore, there is a need to leverage repeated-measures data across multiple time points to examine patterns of mental health and potential recovery or deterioration over time. This evidence gap is especially pronounced in primary care and across its diverse professional groups, where understanding long-term mental health trends is critical for informing early interventions that addresses manpower shortfalls and for guiding strategies to support wellbeing, recruitment, and retention.

Our primary care institution, National University Polyclinics (NUP) is consistently recognized among Singapore’s top employers [15]. Understanding the baseline and evolving mental health profile of NUP employees drawn from repeated-measures screening data will provide actionable insights to sustain positive outcomes and address areas of vulnerability.

Accordingly, this study leveraged routinely collected health screening survey data to examine patterns in the mental health and wellbeing of primary care healthcare professionals across two independent observation windows spanning a six-year period (2018–2025). Given that HADS and GHQ-12 capture overlapping but non-identical constructs and employ different scoring schemes, the two windows are treated as analytically distinct rather than as a single continuous time series. Grounded in the JD-R framework, the primary aim is to identify factors associated with distress that are consistent across both windows despite the transition in screening instrument, rather than to characterise a single continuous pattern of change. As our dataset was collected for operational occupational health screening rather than to test the JD-R model directly, this framework is applied here as a broad organizing lens for interpreting associations rather than as a formally tested theoretical model. We hypothesized that personal demands, such as chronic diseases and medication requirements, along with the high job demands inherent in patient-facing roles, would consistently correlate with higher psychological distress [16]. In contrast, we anticipated that regular exercise would be associated with lower odds of psychological distress, while smoking and alcohol use would be associated with higher odds of psychological distress [17].

## 2. Materials and Methods

### 2.1. Study Design and Population

We conducted a repeated-measures study using annual health screening data from healthcare professionals employed by NUP in Singapore (January 2018–August 2025), comprising two analytically independent observation windows: 2018–2022 (HADS) and 2023–2025 (GHQ-12). Health screenings were held annually, except during the COVID-19 operational peak years of 2020 and 2021 when activities were suspended. All NUP employees across seven large primary care clinics and headquarters were invited to the screening via corporate communications. Participation was voluntary, and response rates were calculated annually as the ratio of attendees to the total eligible workforce to assess representativeness. Only a subset of staff contributed observations at multiple time points; the dataset therefore reflects repeated, incomplete observation of a dynamic workforce rather than systematic follow-up of a fixed cohort.

### 2.2. Data and Variables Measurement

The de-identified health screening dataset included employee demographics (age, sex, race, marital status), occupational information (profession and workplace), and clinical measures obtained on the day of screening: fasting serum glucose and lipid panel, and anthropometrics (height, weight, and blood pressure). Self-reported data covered personal and family history of medical conditions, current use of chronic medications, and lifestyle behaviors (smoking status, alcohol use, physical activity/exercise and their frequencies). Consistent with the JD-R framework, self-reported adherence to age-appropriate cancer screening (e.g., mammography, fecal occult blood testing) was included as a proxy for proactive health-seeking behaviour (a personal resource that may reflect higher self-efficacy and resilience against psychological distress) and health activation (proactive engagement with one’s own health, which may protect against psychological distress). As no validated measure of health activation or health-seeking behaviour were available within the screening dataset, this variable was treated as an exploratory proxy, and its associations with distress outcomes should be interpreted accordingly. More broadly, none of the variables in this dataset were designed as validated measures of JD-R demand or resource constructs; associations reported here are interpreted through this framework as a post hoc organizing lens.

### 2.3. Inclusion and Exclusion Criteria

The health screening was open to all NUP-employed staff, including those on probation. Inclusion criteria comprised all NUP employees at the time of screening who provided consent to the health screening. Participation in blood tests and anthropometric measurements required consent; therefore, records in the dataset had documented consent. From 2022 to 2025, dental staff and dentists within NUP were also included. In 2025, pharmacy and diagnostics staff were included. Exclusion criteria were temporary staff, locum doctors, medical officers rotating through NUP as part of their postings, and family medicine residents still in training.

### 2.4. Outcome Definition

The primary outcome was mental health status, assessed using two validated self-report screening instruments of psychological distress: the Hospital Anxiety and Depression Scale (HADS) and the General Health Questionnaire-12 (GHQ-12) [18]. HADS was administered from 2018 to 2022; GHQ-12 was used from 2023 to 2025. This transition reflected an institutional decision to adopt an instrument better suited to routine, high-throughput occupational screening, given GHQ-12’s brevity, low respondent burden, and simple bimodal scoring with a threshold. HADS is a 14-item instrument developed to screen for anxiety and depression in medically ill populations, comprising two 7-item subscales (Anxiety, HADS-A; Depression, HADS-D), each scored on a 0–3 Likert scale, with subscale scores ranging from 0 to 21. It is sensitive to change over time and useful for monitoring symptom change and treatment response, with good sensitivity and specificity for anxiety and depressive disorders in medical settings and has been validated in primary care [19,20,21,22]. GHQ-12 is a 12-item screening instrument for general psychological distress and minor (non-psychotic) psychiatric morbidity, primarily reflecting anxiety and depressive symptoms [23,24]. It has been widely validated across diverse adult populations and settings, demonstrating good reliability and consistency for screening [25,26]. While not diagnostic, it is effective for identifying individuals at risk who may benefit from further assessment.

For routine staff screening in this study, we applied established cut-offs. For HADS, an abnormal result was defined as a score ≥ 8 on either the HADS-A or HADS-D subscale, consistent with the original scale developers’ threshold for possible caseness and with prior studies using this composite either-subscale definition to identify overall psychological distress [20,27,28]. This threshold was applied as an operational screening flag to prompt individualized follow-up with staff meeting criteria, rather than a diagnostic threshold. For GHQ-12, we used bimodal scoring (0-0-1-1) with a threshold of ≥2 to indicate psychological distress, consistent with an earlier internal validation conducted in 2003 [29,30,31]. These thresholds were used to derive binary outcomes (case vs. non-case) for subsequent analysis.

### 2.5. Data Cleaning and Statistical Analyses

Data cleaning and statistical analysis were performed using Microsoft^®^ Excel^®^ 2016 MSO (Version 2608, Build 16.0.20326.20072, 64-bit; Microsoft Corporation, Redmond, WA, USA), RStudio (version 2026.01.0, Build 392, “Apple Blossom” Release; Posit Software, PBC, Boston, MA, USA) running R (version 4.5.1; R Core Team, R Foundation for Statistical Computing, Vienna, Austria), and SPSS (Version 29; IBM Corp., Armonk, NY, USA). Preliminary data cleaning was performed to remove irrelevant variables and construct new variables. Composite chronic disease variables such as hypertension, hyperlipidemia, diabetes, and pre-diabetes were generated to capture chronic disease status. Staff were classified as having hypertension if they had a Systolic Blood Pressure (SBP) ≥ 140 mmHg, or Diastolic Blood Pressure (DBP) ≥ 90 mmHg, or a prior diagnosis of hypertension. Hyperlipidemia was based on LDL ≥ 4.1 mmol/L, triglycerides ≥ 2.3 mmol/L, self-reported high cholesterol, or use of cholesterol-lowering medications. Staff were classified as having diabetes if their fasting glucose levels were ≥7 mmol/L or if a prior diagnosis of diabetes was reported. Staff were classified as having pre-diabetes if their fasting glucose levels were between 6.1 mmol/L and 6.9 mmol/L or had a prior diagnosis of pre-diabetes, without diagnosis of diabetes. A composite variable for cancer screening was created to indicate whether staff had undergone any form of screening, such as cervical cancer screening, breast screening mammogram, or colonoscopy. The family history of chronic disease variable captured staff who reported having immediate family members with conditions such as hypertension, diabetes, or hyperlipidemia. The medication variable indicated whether the staff were on prescribed long-term medications. The ‘significant past history’ variable was derived from a self-report item asking whether the staff had significant past medical history (yes/no), with free-text examples requested when ‘yes’ was selected; ‘significant’ reflects each participants’ own assessment rather than a severity threshold applied by the research team. Free-text entries (Supplementary Table S1) were reviewed by the research team solely for data quality and coding consistency, not for clinical adjudication of severity. We identified 12 missing or unknown data points (0.36%). Given this negligible rate, we utilized complete-case analysis for the primary models. To ensure robustness, we performed a sensitivity analysis comparing these results against a “conservative imputation” approach (where missing clinical conditions were coded as “No”) and found no significant divergence in the direction or magnitude of effect sizes.

Statistical significance was assessed at *p* < 0.05 using a two-tailed test and descriptive statistics were used to characterize the sample. Categorical variables were reported as counts and percentages, and continuous variables that were not normally distributed were expressed as median and inter-quartile range. For participants with multiple screenings, person-level variables presented in the descriptive summary of the overall study population were defined using the most recent available data. Group differences were examined using Pearson’s chi-square tests for categorical variables (with Fisher’s exact test applied when expected cell counts were <5) and Mann–Whitney U tests for non-normally distributed continuous variables. When overall *p* < 0.05 for comparisons with >2 groups, post hoc pairwise differences were tested using Tukey’s HSD.

The mixed-effects logistic regression models for HADS and GHQ-12 constituted the primary analyses. Linear mixed-effects models and the frequent-attender subgroup analysis were conducted as secondary and exploratory sensitivity analyses to assess robustness, respectively; findings emerging only within these analyses should be interpreted as exploratory rather than confirmatory. Covariates included in the multivariable models were selected a priori based on the JD-R framework and clinical relevance, and were not modified based on the results of the bivariate analyses. To account for within-person clustering among staff with repeated screenings across years, we fitted mixed-effects logistic regressions for abnormal GHQ-12 and HADS scores separately, using each visit’s own measured covariate values (rather than a value carried forward from another visit), including a random intercept for each participant and adjusting for all selected predictors. Screening year was included as a fixed effect in all models. Results from logistic models were reported as odds ratios (ORs) with 95% confidence intervals (CIs). As part of our sensitivity analyses, we fitted linear mixed-effects regression models to examine factors associated with numerical GHQ-12 and HADS scores, adjusting for the same predictors. Continuous outcomes from linear mixed models are reported as regression coefficients (β) with 95% CIs. We also performed a prespecified sensitivity analysis restricted to staff with ≥3 screening visits to evaluate robustness of associations in a subgroup with more extensive repeated measures data.

Multicollinearity among covariates was assessed using the Generalised Variance Inflation Factor (GVIF), with all factors satisfying the pre-specified threshold of GVIF < 5 (maximum GVIF = 3.26, for professional category). This assessment addresses multicollinearity specifically and does not by itself establish correct model specification. Model fit was evaluated using the Akaike Information Criterion (AIC) and Bayesian Information Criterion (BIC). The explanatory power of each final model was characterised using marginal R^2^ (variance explained by fixed effects alone) and conditional R^2^ (variance explained by both fixed and random effects combined), calculated using the method of Nakagawa and Schielzeth. For the primary logistic mixed-effects models, the HADS model yielded an AIC of 2027.5, BIC of 2212.3, marginal R^2^ of 0.123, and conditional R^2^ of 0.518; the GHQ-12 model yielded an AIC of 1417.8, BIC of 1606.3, marginal R^2^ of 0.175, and conditional R^2^ of 0.565. For the linear mixed-effects models, the HADS model yielded an AIC of 9989.1, BIC of 10,179.4, marginal R^2^ of 0.086, and conditional R^2^ of 0.621; the GHQ-12 model yielded an AIC of 6374.2, BIC of 6568.1, marginal R^2^ of 0.098, and conditional R^2^ of 0.415. For the subgroup logistic mixed-effects models restricted to frequent attenders, the HADS model yielded an AIC of 1274.0, BIC of 1444.9, marginal R^2^ of 0.135, and conditional R^2^ of 0.636; the GHQ-12 model yielded an AIC of 983.6, BIC of 1155.2, marginal R^2^ of 0.178, and conditional R^2^ of 0.556. Across all models, marginal R^2^ values were modest (range 0.086–0.178), reflecting the multifactorial and partially unmeasured determinants of psychological distress inherent to observational data. Conditional R^2^ values were substantially higher (range 0.415–0.636), indicating that the random effects captured meaningful between-individual heterogeneity and affirming the appropriateness of the mixed-effects modelling approach for repeated-measures data of this kind.

## 3. Results

A total of 1260 staff with 3307 health screening visits were included in the analysis. The median age of healthcare staff was 37 (IQR 18), with majority being females (86.3%) and ethnically Chinese (72.7%) (Table 1). Almost half were married (49.3%). The largest numbers of staff attendees were from headquarters (16.2%) and clinic E (16.3%); ancillary (*n* = 448, 35.6%) formed the biggest group who attended health screenings, followed by nursing (*n* = 318, 25.2%), and administrative staff (*n* = 225, 17.9%). The eligible workforce and participation rate for each annual health screening were presented in Table 1; participation declined from 72.9% in 2018 to 49.3% in 2025 despite growth in the eligible workforce over the same period. Comparing the characteristics of attendees at each annual screening as independent cross-sectional snapshots, sex (*p* = 0.88) and race (*p* = 0.11) distributions were similar across years. In contrast, marital status (*p* < 0.001), institution (*p* < 0.001) and job class (*p* < 0.001) differed significantly across the years, partly attributed to increasing number of participants “preferring not to disclose” their marital status, Clinic B’s onboarding into screening programme from 2022 onwards, and inclusion of dental staff from 2022, and pharmacy and diagnostics staff for screening in 2025. Across the 6 health screenings across the years, 32.2% of staff attended once, 24.8% attended twice, 16.1% attended thrice, 10.7% attended four times, 7.3% attended five times, and 8.8% attended all health screening events.

Across 3307 screening visits completed by 1260 healthcare staff, the median BMI was 23.0 kg/m^2^ (Table 2). Hypertension was recorded or self-reported in 11.3% of visits, diabetes in 4.2%, and pre-diabetes in 2.9%. Hyperlipidemia (self-reported or confirmed by blood tests) was noted in 48.6% of visits, and 17.8% reported other significant past medical history. Long-term medication use was reported in 10.4% of visits; 1.9% reported active smoking; 24% reported regular alcohol use; and 42.5% reported regular exercise. A family history of chronic disease was recorded in 79.3% of visits, and 58.5% reported having undergone at least one cancer screening. Because multiple visits were completed by the same individuals, these figures reflect visit-level proportions rather than unique person-level prevalence.

**Figure 1:**
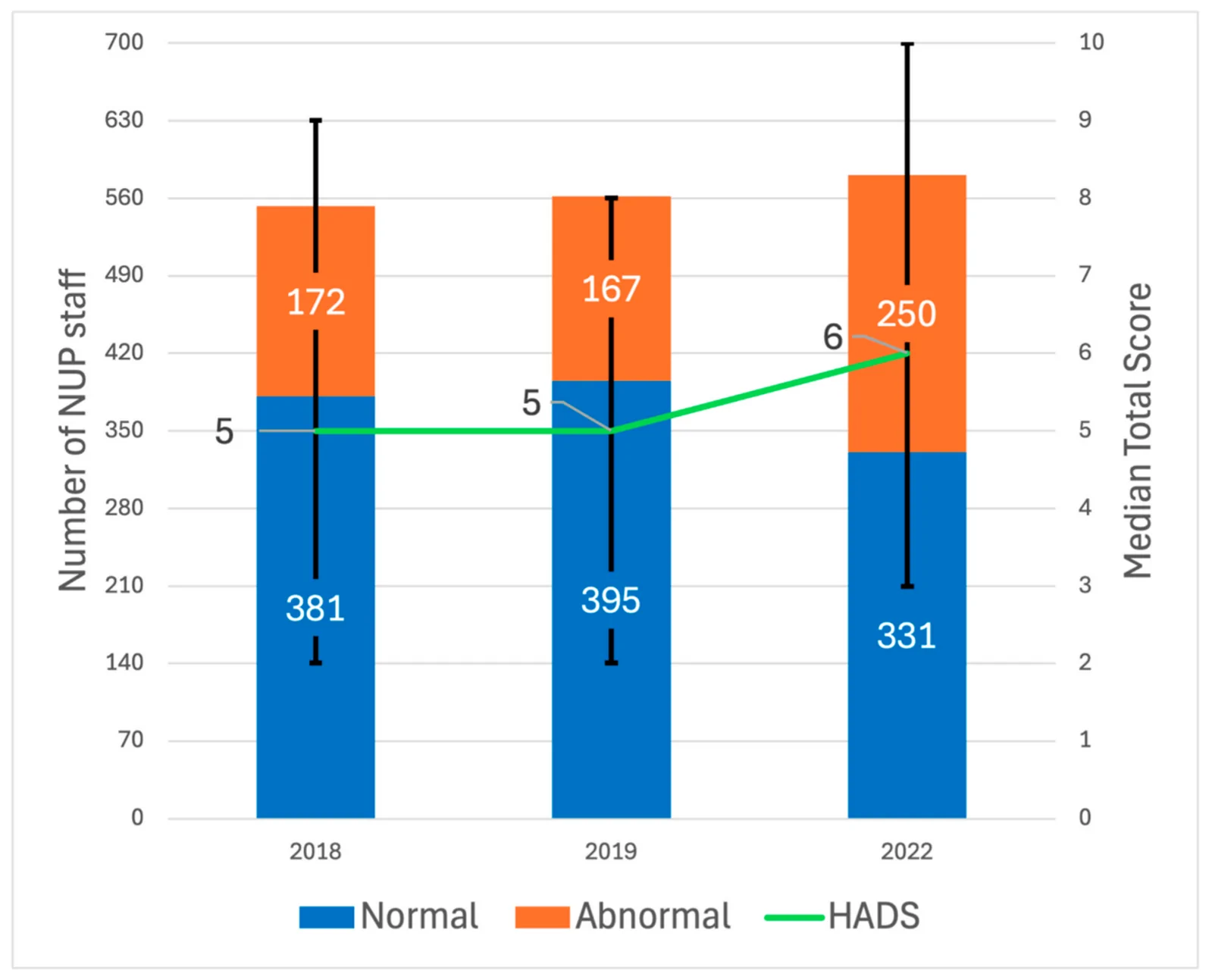
Primary care staff with HADS scores from 2018 to 2022.

We noted a significant increase (*p* < 0.001) in abnormal HADS scores from 2018 (*n* = 172, 29.2%) to 2022 (*n* = 250, 42.4%) (Table 2, Figure 1). Staff with abnormal HADS scores had a significantly higher median BMI (*p* = 0.018) and were more likely to smoke (*p* = 0.006) and drink alcohol regularly (*p* = 0.001). They were less likely to exercise regularly (*p* = 0.006) or attend cancer screening (*p* < 0.001). There were no significant differences in the prevalence of diabetes, hypertension, hyperlipidemia, newly diagnosed chronic conditions, long-term medication use, significant past medical history, or a family history of chronic disease between staff with normal versus abnormal HADS (*p* > 0.05 for all).

From 2018 to 2022, our mixed-effects logistic regression indicated that, compared with 2018, 2022 was associated with significantly higher odds for abnormal HADS score (OR 2.28, 95% CI 1.64–3.17, *p* < 0.001), as was regular alcohol use (OR 1.56, 95% CI 1.05–2.31, *p* = 0.027) (Table 3). Significant differences in HADS scores were observed across professions (*p* = 0.019); medical (OR 0.39, 95% CI 0.21–0.74, *p* = 0.004) and nursing staff (OR 0.56, 95% CI 0.37–0.87, *p* = 0.009) had lower odds of abnormal HADS scores compared with ancillary staff. However, in Tukey HSD-adjusted post hoc comparisons, significant pairwise differences were observed only for medical vs. ancillary (*p* = 0.03); other pairwise comparisons were not statistically significant after adjustment. In contrast, regular exercise (OR 0.72, 95% CI 0.529–0.991, *p* = 0.044) and each additional year of age was associated with lower odds (OR 0.967, 95% CI 0.948–0.987, *p* = 0.001) of an abnormal HADS score.

In our sensitivity analyses employing linear mixed-effects regression for 2018–2022, HADS scores were significantly higher in year 2022 versus 2018 (1.40 points, 95% CI 0.94 to 1.85, *p* < 0.001), among smokers (1.93 points, 95% CI 0.15 to 3.71, *p* = 0.034), and those with significant past medical history (0.68 points, 95% CI 0.00 to 1.35, *p* = 0.05) (Table S2). By contrast, lower HADS scores were significantly associated with older age (−0.07 points per year; 95% CI, −0.10 to −0.04; *p* < 0.001), working in the medical (−1.78 points; 95% CI, −2.86 to −0.69; *p* = 0.001) or nursing profession (−1.02 points; 95% CI, −1.77 to −0.27; *p* = 0.008) versus ancillary staff; pairwise comparisons showed significant differences only between medical and ancillary (*p* = 0.01), and medical and administrative (*p* = 0.003). HADS scores were also significantly lower among those who participated in regular exercise (−0.57 points; 95% CI, −1.04 to −0.09; *p* = 0.019) versus never exercising. Regular alcohol use was not associated with increase in HADS scores (*p* = 0.165).

In a sensitivity analysis restricted to primary care staff who were frequent attenders (≥3 attendances), 2022 was associated with higher odds of abnormal HADS scores (OR 2.82, 95% CI 1.81–4.39, *p* < 0.001) compared to 2018. Regular exercise (OR 0.59, 95% CI 0.37–0.94, *p* = 0.03) and age remained associated with lowered odds of abnormal HADS scores (OR 0.959 per year, 95% CI 0.928–0.99, *p* = 0.01) (Table S3). Factors that were previously non-significant became significant in this subgroup, including participation in cancer screening (OR 0.34, 95% CI 0.17–0.67, *p* = 0.002), and BMI (OR 1.08 per unit, 95% CI 1.01–1.14, *p* = 0.011).

**Figure 2:**
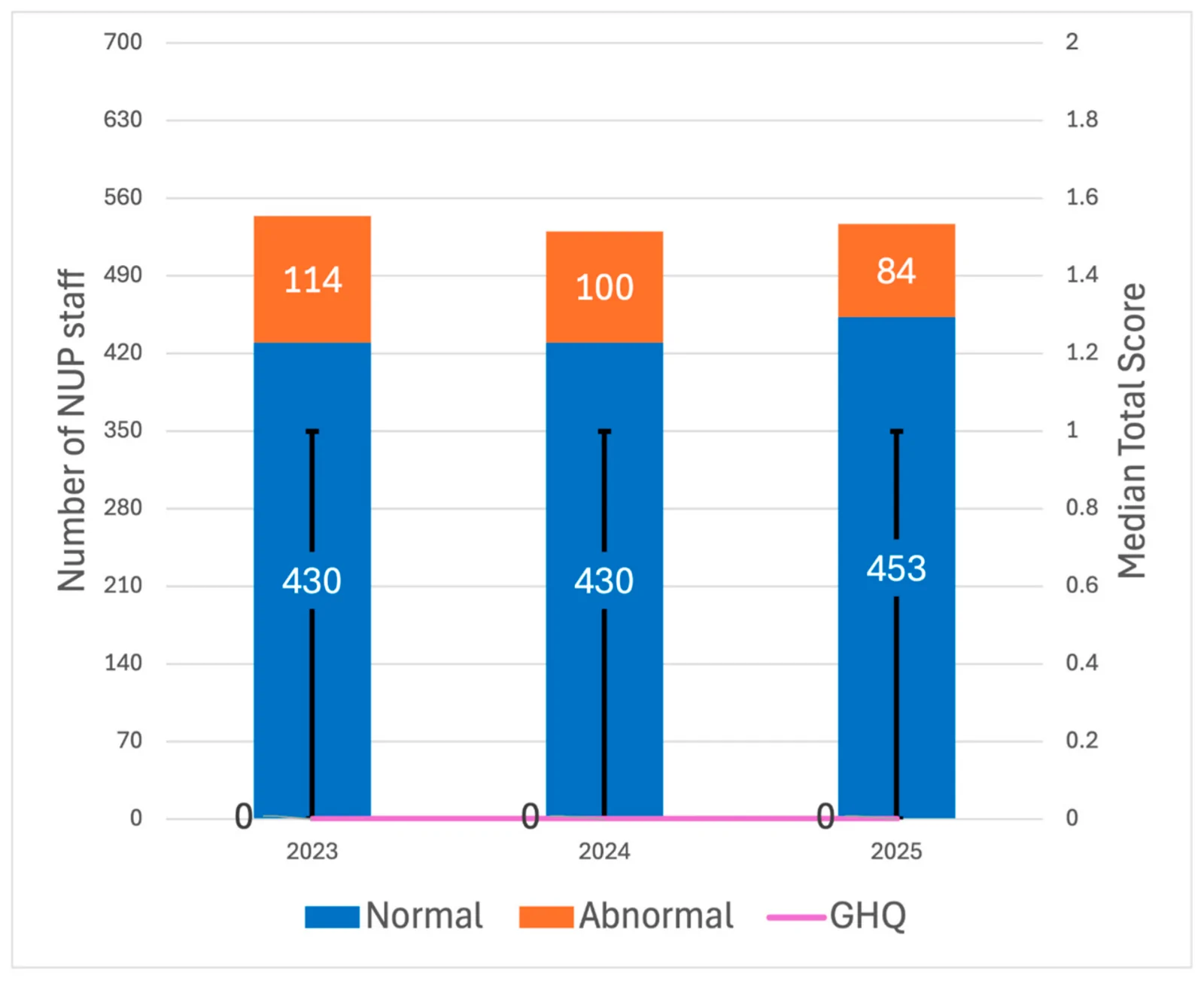
Primary care staff with GHQ scores from 2023 to 2025.

The proportion of staff with abnormal GHQ-12 scores decreased from 2023 (*n* = 114, 38.3%) to 2025 (*n* = 84, 28.2%) (*p* = 0.077), but this was not statistically significant (Table 2, Figure 2). Between 2023 and 2025, abnormal GHQ-12 scores were significantly more common among staff with significant past medical history (*p* = 0.035) and a family history of chronic disease (*p* = 0.008), those not on long-term medications (*p* = 0.009) and active smokers (*p* < 0.001). Abnormal GHQ-12 scores were also significantly more common among staff who did not exercise regularly (*p* < 0.001) and those who did not participate in regular cancer screening (*p* < 0.001). There were no significant differences in diabetes, hypertension, hyperlipidemia, newly diagnosed chronic conditions, regular alcohol use, or median BMI between staff with normal versus abnormal GHQ-12 (*p* > 0.05 for all).

From our multivariate mixed effects logistic regression, having a family history of chronic disease (OR 2.22, 95% CI 1.28 to 3.87, *p* = 0.005), and active smoking (OR 5.79, 95% CI 1.75 to 19.10, *p* = 0.004) were associated with higher odds of an abnormal GHQ-12 score (Table 4). Race was found to significantly influence GHQ-12 scores (*p* = 0.031), with those being of Other race versus Chinese (OR 2.91, 95% CI 1.22 to 6.94, *p* = 0.016) associated with higher odds of an abnormal GHQ-12. However, pairwise comparisons were not significant after post-hoc adjustments (*p* > 0.05 for all). In contrast, increasing age (OR 0.955 per year, 95% CI 0.928 to 0.983, *p* = 0.002) and regular exercise (OR 0.36, 95% CI 0.23 to 0.55, *p* < 0.001) were associated with lower odds of an abnormal GHQ-12 score.

Our linear mixed-effects model indicated that higher GHQ-12 scores were associated with a family history of chronic disease (0.26 points, 95% CI 0.03 to 0.49, *p* = 0.029) and active smoking (2.19 points, 95% CI 1.50 to 2.89, *p* < 0.001) (Table S4). Conversely, lower GHQ-12 scores were observed among staff who exercised regularly (−0.41 points, 95% CI −0.59 to −0.22, *p* < 0.001).

Our sensitivity analysis on primary care staff who were frequent attenders showed that for 2023–2025, active smoking was associated with higher odds of abnormal GHQ-12 scores (OR 6.59, 95% CI 1.42–30.59, *p* = 0.016), while self-reported regular exercise was associated with lower odds (OR 0.35, 95% CI 0.21–0.59, *p* < 0.001), consistent with the main analysis (Table S5). However, some factors that were significant in the main analysis were not significant among frequent attenders: age (*p* = 0.109) and family history of chronic diseases (*p* = 0.096).

## 4. Discussion

This multi-year analysis of routine primary care staff health screenings identified occupational and behavioural factors associated with psychological distress that were consistent across two independently analysed observation windows. Within the HADS window (2018–2022), abnormal scores were significantly higher in 2022 than in 2018, suggesting elevated distress during the post-acute pandemic period. Within the GHQ-12 window (2023–2025), a non-significant downward trend was observed, though the use of a different instrument precludes direct comparison with the earlier period. Across both windows, older age and regular exercise were consistently associated with lower odds of distress, while active smoking was associated with higher odds, consistent with findings from frequent attender subgroups. In the HADS window, staff in medical roles had lower distress than ancillary staff, and regular alcohol use was associated with higher HADS scores. In the GHQ-12 window, a family history of chronic disease was associated with higher distress. Together, these findings point to modifiable behavioural factors (exercise, smoking) and contextual factors (profession, family history) as areas warranting further attention in occupational health planning.

These patterns may reflect the evolving operational context of primary care, with cumulative post-COVID-19 stressors potentially contributing to elevated distress in 2022. The elevated HADS scores observed in 2022 are broadly consistent with contemporary studies conducted during the same period [8,12,13,32], which captured residual strain from service disruptions, workload fluctuations, and sustained uncertainty. The numerical, non-significant downward trend in GHQ-12 scores from 2023 to 2025 does not constitute evidence of improvement and should not be interpreted as recovery; it may reflect stabilization of care delivery coinciding with Singapore’s transition to DORSCON Green in February 2023 [33], but the change in screening instrument and lack of statistical significance mean this observation cannot be treated as a direct continuation of, or improvement upon, the HADS-window findings. The concept of burnout as a cumulative, lagging phenomenon rather than an acute stress reaction is well-established in the literature, and our findings are broadly consistent with this model. Although some of the studies cited above overlapped with our survey period, they used different instruments to assess distress or burnout, limiting direct comparability.

While this study was not able to measure psychological distress in years 2020–2021, longitudinal data from hospital workers tracked repeatedly through 2023 suggest that distress and burnout did not follow a monotonically rising trend tied to case load, but changed dynamically in ways not fully captured by studies ending in 2021 [34]. Studies during this time reported that distress levels remained above pre-pandemic benchmarks, consistent with incomplete psychological recovery even in periods of relative epidemiological calm [35]. This sustained stress has been linked to absenteeism, turnover, and medical errors, suggesting that distress may persist well beyond the acute phase of health emergencies [36]. As noted above, the non-significant downward trend observed in GHQ-12 scores from 2023 to 2025 does not constitute evidence of improvement and should not be interpreted as recovery.

We observed consistently lower psychological distress among staff in clinical roles (medical, dental, nursing) compared with administrative and administrative staff. The prior literature had proposed several contributing factors, including differences in work autonomy, peer support, remuneration, and professional identity [32,37,38,39,40], though these factors were not directly measured in our study and their relevance to our findings cannot be confirmed. The finding that pairwise post-hoc significance was retained only for the medical versus ancillary comparisons suggests this contrast merits further investigation.

The association between older age and lower odds of psychological distress persisted across both cohorts, both instruments, and sensitivity analyses. Several non-exclusive mechanisms may explain this. Older workers may possess greater emotional regulation capacity and better coping strategies accumulated over their working lives [41]. Research has demonstrated that younger adults report greater psychological distress alongside fewer emotion regulation resources compared to middle-aged and older adults, suggesting that age-related advantages in distress are at least partly mediated through accumulated psychological skills [42]. Career stage may also matter: senior staff may have clearer professional identities, more established collegial networks, and less vulnerability to the occupational uncertainty that disproportionately affects junior staff during organizational disruptions [43]. Lastly, a ‘healthy worker’ effect cannot be excluded: workers who have remained in a demanding primary care role for many years may represent a self-selected resilient subgroup, with psychologically vulnerable individuals having left the workforce over time, inflating the apparent association between older age and lower odds of distress.

Several behavioral, social, and lifestyle factors identified in this study were associated with psychological distress. Active smoking was associated with higher distress, consistent with international evidence [44]. The causal direction is complex and likely bidirectional: smoking may exacerbate anxiety and depressive symptoms, while distress may drive nicotine use as a coping mechanism. Unmeasured confounders such as financial strain, family challenges, or psychiatric comorbidity may contribute to both smoking behaviour and psychological distress [45,46].

Regular exercise showed a consistent association with lower odds of psychological distress across both cohorts, surviving sensitivity analyses among frequent attenders. The mechanisms are likely multifactorial, encompassing neurobiological pathways (e.g., neurotransmitter modulation) and psychological benefits including enhanced self-esteem, coping, and distraction [47,48,49]. Stronger positive association in our subgroup analysis could reflect true effect modification tied to health-seeking behaviour (staff who both attend screenings and exercise may benefit more), or reduced confounding among frequent attenders (e.g., better sleep, healthier dietary choices). Importantly, the relationship between exercise and distress is likely bidirectional: distress is itself a known barrier to initiating or sustaining physical activity [50,51]. Organizational interventions designed to reduce structural barriers to exercise, such as protected time, on-site facilities, and subsidised fitness programmes, may therefore have genuine mental health co-benefits [52], particularly for ancillary and administrative staff with more limited access to informal wellness resources.

Regular alcohol use was associated with higher odds of abnormal HADS scores in our logistic model but not in the linear model or the GHQ-12 cohort, suggesting a threshold rather than a dose–response relationship, or genuine secular change in alcohol use patterns across the two time periods. This warrants systematic screening within future occupational health programmes.

The association between family history of chronic disease and higher odds of abnormal GHQ-12 scores was not explained by our measured variables. The prior literature had proposed several possible contributing mechanisms, including caregiving burden, financial pressures, and health-related anxiety [53,54,55,56], though these were not assessed in the present study. This association was not significant in the earlier HADS window; we cannot determine from the current data whether this reflects a genuine difference in periods, workforce ageing, or chance, and this should be treated as hypothesis-generating rather than explanatory.

Findings from the frequent-attender subgroup should be interpreted as exploratory rather than confirmatory, given that this subgroup itself may represent a selected, more health-engaged group rather than a random sample of the study population. Higher BMI was associated with greater odds of abnormal HADS scores in this subgroup (*p* = 0.011) but not in the main analysis. Frequent attenders may represent a more health-conscious and self-monitoring subgroup, in whom BMI-related health anxiety or weight-related stigma in healthcare workplaces may more consistently translate into measurable psychological distress [57,58], though these mechanisms were not directly assessed in our study. Alternatively, the increased statistical power afforded by repeated observations within this subgroup may explain why this association reached significance only here; this remains hypothesis-generating rather than evidence of a true association obscured in the full heterogeneous cohort (*p* = 0.075). Cancer screening participation was inversely associated with abnormal HADS scores among frequent attenders (*p* = 0.002), though not reaching significance in primary models. As noted in Methods, cancer screening was included as an exploratory, unvalidated proxy for health activation and health-seeking behaviour [59,60]; this association should be interpreted with corresponding caution rather than as evidence of a specific underlying psychological mechanism.

In bivariate analysis, staff not on long-term medications were paradoxically more likely to have abnormal GHQ-12 scores, a finding running counter to the clinical expectation that chronic disease burden would be associated with greater distress. This association did not persist in any of our multivariate mixed-effects models after adjustment indicating that it reflects confounding rather than a genuine independent effect. Staff on long-term medications may have established therapeutic relationships and structured treatment pathways providing a degree of psychological containment [61]. Residual confounding by age (older staff being both more likely to use medications and less likely to report distress) may also partially explain this pattern. We treat this as a spurious bivariate association and draw no inferential conclusions from it.

A key measurement consideration is the transition from usage of HADS (2018–2022) to GHQ-12 (2023–2025), which is why the two periods are analysed as independent repeated-measures windows rather than a single continuous series. Although both screen for psychological distress, they capture overlapping but non-identical constructs and use different scoring schemes and thresholds, limiting strict longitudinal comparability and likely contributing to differences in distress proportions across these periods. As HADS was phased out in 2022, future surveillance will rely on GHQ-12, mitigating ongoing comparability concerns. GHQ-12 is well-suited for primary care healthcare professionals because it is brief and low-burden for busy staff, has strong reliability and broad validation across occupational and non-psychiatric settings, focuses on general (non-psychotic) distress relevant to diverse roles, uses simple bimodal scoring with a consistent threshold (internally validated at our institution), and yields a single summary measure that supports efficient, repeated annual monitoring and straightforward triage pathways [24,25,62].

One of the key strengths of this study was its large, multi-year dataset integrating objective clinical measures with standardized screening of psychological distress with minimal missing data. Its appropriate use of mixed-effects models with participant-level random intercepts to account for repeated measures and respondent heterogeneity aided in the robustness of findings within each observation window. Predefined cut-offs and multicollinearity checks support construct validity and model integrity.

This study has several limitations. First, it is important to note that this dataset does not represent a conventional longitudinal cohort systematically followed over time: participation was voluntary, and attendance varied substantially by year. Mixed-effects models appropriately account for within-person correlation among the repeated observations, but this analytic accommodation does not by itself confer the properties of a systematically followed cohort. A comparison of attendee characteristics across each annual screening found sex and race distributions stable across time, providing some reassurance against gross demographic selection effects on these characteristics. However, marital status, institution, and job class distributions differed significantly across the years, indicating the attending population was not static in composition; this is attributable in part to identifiable structural and reporting changes to the screening programme, rather than selection on distress-related characteristics per se, though the possibility of the latter cannot be excluded. Relatedly, and most critically for the interpretation of temporal trends, participation declined substantially across the study period—from 72.9% in 2018 to 49.3% in 2025. This monotonic decline introduces a plausible and important source of selection bias that is directly relevant to the observed trends in distress scores. If psychologically distressed staff are disproportionately likely to disengage from voluntary wellness screening over time—a pattern well-documented in occupational health settings—then the apparent non-significant decline in GHQ-12 scores from 2023 to 2025 may partly or wholly reflect a progressively healthier and more engaged attender pool rather than genuine improvement in the workforce’s mental health. This possibility cannot be ruled out and means that the downward trend in the GHQ-12 window should be interpreted with particular caution. Individual-level data on non-attenders were unavailable, precluding formal comparison of attenders and non-attenders on observable characteristics. Accordingly, our findings should be interpreted as reflecting the psychological health of staff who engaged with occupational wellness screening, rather than as prevalence estimates for the entire NUP workforce. Without data on non-participants, the direction of any resulting selection bias cannot be established. Self-reported behaviour (smoking, alcohol use, exercise, cancer screening) are susceptible to social desirability and recall bias, likely leading to underreporting; if misreporting occurs at similar rates across groups, real between-group differences may be attenuated. The “significant past history” variable combines a clinically heterogeneous range of self-reported conditions, limiting interpretability of associations involving this variable, and findings should not be taken to imply a specific severity or chronicity of underlying disease. HADS and GHQ-12 are screening tools, not diagnostic instruments, and the abnormal scores and cutoffs reflect risk rather than confirmed disorder. Visit-level proportions reflect encounters rather than unique individuals, and repeated attendance by some staff can influence counts. The retrospective design constrained variable selection; we lacked data on workload, shift schedules, caregiving responsibilities, sleep, financial status and burnout, which can directly impact psychological distress. While pairwise comparisons were adjusted using Tukey HSD correction, small subgroup sizes, notably the “Other” race category, may yield unstable estimates that should be treated as hypothesis-generating. Continuous predictors (age, BMI) were entered as linear terms without formally testing for nonlinearity; while a linear association between age and psychological distress is well supported in the literature, we cannot exclude the possibility of a non-monotonic relationship between BMI and distress that a linear term would not capture. Lastly, the core JD-R constructs (such as workload, job control, emotional demands, supervisor and peer support) were not directly measured; future occupational health surveys would benefit from incorporating brief, validated JD-R aligned measures alongside existing screening instruments, which would allow the framework to be tested prospectively rather than applied interpretively.

## 5. Conclusions

The consistent identification between physical inactivity, younger age and psychological distress across both independently analysed observation windows, alongside period specific findings—ancillary occupation and alcohol use within the HADS window, and active smoking and family history of chronic disease within the GHQ-12 window—suggests potential areas for consideration in organisational planning, though the effectiveness of any specific intervention was not evaluated in this study. Healthcare organisations may consider candidate structural approaches, such as protected time for physical activity, formal peer support programmes with outreach to non-clinical staff, and regular psychological health surveillance as a routine component of occupational health. The numerical, non-significant reduction in GHQ-12 scores from 2023 to 2025 should not be taken to indicate resolution of occupational distress; sustained monitoring using a consistent instrument will be essential for detecting meaningful change over time. The prior literature has linked unaddressed occupational distress among healthcare workers to downstream effects on patient safety and care quality; while this relationship was not directly assessed in the present study, it underscores staff mental health as a systems-level concern warranting continued attention alongside individual welfare.

## Figures and Tables

**Table 1:** Descriptive statistics and demographics of staff health screening visits, by year (3307 visits; 1260 unique staff).

Characteristic	Total	2018	2019	2022	2023	2024	2025	*p*
	*n* = 1260	*n* = 553	*n* = 562	*n* = 581	*n* = 544	*n* = 530	*n* = 537	
Eligible workforce		759	776	863	973	1002	1089	
Participation rate		72.9%	72.4%	67.3%	55.9%	52.9%	49.3%	
Age, Mean (SD)	40 (12)	41 (12)	41 (12)	40 (12)	40 (11)	40 (12)	40 (11)	
Age, Median (IQR, Min–Max)	37 (30–48, 20–74)	40 (31–51, 20–72)	40 (31–52, 21–70)	38 (32–49, 20–73)	38 (31–48, 20–74)	37 (31–48, 20–70)	37 (31–47, 21–71)	0.07 ^a^
*Sex (n, %)*								0.88 ^b^
Female	1087 (86.3)	485 (87.7)	499 (88.8)	511 (88.0)	471 (86.6)	461 (87.0)	466 (86.8)	
Male	173 (13.7)	68 (12.3)	63 (11.2)	70 (12.0)	73 (13.4)	69 (13.0)	71 (13.2)	
*Race (n, %)*								0.11 ^b^
Chinese	916 (72.7)	394 (71.2)	412 (73.3)	430 (74.0)	394 (72.4)	390 (73.6)	388 (72.3)	
Indian	101 (8.0)	52 (9.4)	45 (8.0)	43 (7.4)	43 (7.9)	39 (7.4)	44 (8.2)	
Malay	164 (13.0)	57 (10.3)	58 (10.3)	78 (13.4)	77 (14.2)	72 (13.6)	75 (14.0)	
Others	79 (6.3)	50 (9.0)	47 (8.4)	30 (5.2)	30 (5.5)	29 (5.5)	30 (5.6)	
*Marital Status (n, %)*								<0.001 ^b^
Married	621 (49.3)	344 (62.2)	370 (65.8)	390 (67.1)	354 (65.1)	257 (48.5)	212 (39.5)	
Single	325 (25.8)	178 (32.2)	165 (29.4)	163 (28.1)	163 (30.0)	136 (25.7)	92 (17.1)	
Others	314 (24.9)	31 (5.6)	27 (4.8)	28 (4.8)	27 (5.0)	137 (25.8)	233 (43.4)	
*Institution (n, %)*								<0.001 ^b^
Clinic A	180 (14.3)	91 (16.5)	90 (16.0)	85 (14.6)	94 (17.3)	82 (15.5)	86 (16.0)	
Clinic B	116 (9.2)	0 (0.0)	0 (0.0)	57 (9.8)	66 (12.1)	59 (11.1)	62 (11.5)	
Clinic C	164 (13.0)	89 (16.1)	80 (14.2)	67 (11.5)	65 (11.9)	58 (10.9)	71 (13.2)	
Clinic D	137 (10.9)	70 (12.7)	74 (13.2)	64 (11.0)	54 (9.9)	58 (10.9)	46 (8.6)	
Clinic E	205 (16.3)	115 (20.8)	107 (19.0)	105 (18.1)	88 (16.2)	76 (14.3)	77 (14.3)	
Clinic F	135 (10.7)	69 (12.5)	85 (15.1)	72 (12.4)	52 (9.6)	47 (8.9)	60 (11.2)	
Clinic G	119 (9.4)	44 (8.0)	54 (9.6)	41 (7.1)	49 (9.0)	49 (9.2)	50 (9.3)	
Headquarters	204 (16.2)	75 (13.6)	72 (12.8)	90 (15.5)	76 (14.0)	101 (19.1)	85 (15.8)	
*Job Class (n, %)*								<0.001 ^b^
Administrative	225 (17.9)	99 (17.9)	86 (15.3)	79 (13.6)	93 (17.1)	90 (17.0)	95 (17.7)	
Allied Health	78 (6.2)	35 (6.3)	43 (7.7)	32 (5.5)	42 (7.7)	37 (7.0)	35 (6.5)	
Ancillary	448 (35.6)	194 (35.1)	190 (33.8)	241 (41.5)	199 (36.6)	209 (39.4)	173 (32.2)	
Medical	141 (11.2)	69 (12.5)	67 (11.9)	67 (11.5)	58 (10.7)	55 (10.4)	42 (7.8)	
Nursing	318 (25.2)	156 (28.2)	176 (31.3)	162 (27.9)	152 (27.9)	139 (26.2)	142 (26.4)	
Others	50 (4.0)	0 (0.0)	0 (0.0)	0 (0.0)	0 (0.0)	0 (0.0)	50 (9.3)	
*Frequency of screening (n, %)*								
1	406 (32.2)							
2	313 (24.8)							
3	203 (16.1)							
4	135 (10.7)							
5	92 (7.3)							
6	111 (8.8)							

^a^ Kruskal–Wallis test. ^b^ Chi-square test.

**Table 2:** Bivariate analysis of staff health screening visits by HADS and GHQ status (normal and abnormal).

Characteristic	Total	HADS < 8 (Normal)	HADS ≥ 8 (Abnormal)	*p*	GHQ < 2 (Normal)	GHQ ≥ 2 (Abnormal)	*p*
	*n* = 3307	*n* = 1107	*n* = 589		*n* = 1313	*n* = 298	
*Year*, *n* (%)				<0.001 ^b^			0.077 ^b^
2018	553 (16.7)	381 (34.4)	172 (29.2)				
2019	562 (17.0)	395 (35.7)	167 (28.4)				
2022	581 (17.6)	331 (29.9)	250 (42.4)				
2023	544 (16.4)				430 (32.7)	114 (38.3)	
2024	530 (16.0)				430 (32.7)	100 (33.6)	
2025	537 (16.2)				453 (34.5)	84 (28.2)	
BMI, Median (IQR)	23.0 (21.0–27.0)	23.0 (21.0–27.0)	24.0 (21.0–28.0)	0.018 ^a^	23.0 (20.0–27.0)	24.0 (20.2–28.0)	0.269 ^a^
*Diabetes, n (%)*				0.155 ^b^			0.665 ^b^
Diabetes	140 (4.2)	62 (5.6)	27 (4.6)		44 (3.4)	7 (2.3)	
Pre-Diabetes	95 (2.9)	41 (3.7)	13 (2.2)		33 (2.5)	8 (2.7)	
No Diabetes	3072 (92.9)	1004 (90.7)	549 (93.2)		1236 (94.1)	283 (95.0)	
*Hypertension, n (%)*				0.161 ^b^			0.642 ^b^
No	2934 (88.7)	972 (87.8)	503 (85.4)		1187 (90.4)	272 (91.3)	
Yes	373 (11.3)	135 (12.2)	86 (14.6)		126 (9.6)	26 (8.7)	
*Hyperlipidemia, n (%)*				0.662 ^b^			0.257 ^b^
No	1699 (51.4)	548 (49.5)	285 (48.4)		697 (53.1)	169 (56.7)	
Yes	1608 (48.6)	559 (50.5)	304 (51.6)		616 (46.9)	129 (43.3)	
Newly Diagnosed Diabetes during health screening, *n (%)*	50 (1.5)	22 (2.0)	10 (1.7)	0.677 ^b^	13 (1.0)	5 (1.7)	0.355 ^c^
Newly Diagnosed Pre-Diabetes during health screening, *n (%)*	66 (2.0)	26 (2.3)	10 (1.7)	0.376 ^b^	24 (1.8)	6 (2.0)	0.813 ^b^
Newly Diagnosed Hypertension during health screening, *n (%)*	135 (4.1)	40 (3.6)	27 (4.6)	0.329 ^b^	55 (4.2)	13 (4.4)	0.893 ^b^
Newly Diagnosed Hyperlipidemia during health screening, *n (%)*	1102 (33.3)	405 (36.6)	211 (35.8)	0.756 ^b^	401 (30.5)	85 (28.5)	0.493 ^b^
*Long-term Medication Use, n (%)*				0.60 ^b^			0.009 ^b^
No	2962 (89.6)	983 (88.8)	518 (87.9)		1179 (89.8)	282 (94.6)	
Yes	345 (10.4)	124 (11.2)	71 (12.1)		134 (10.2)	16 (5.4)	
*Significant Past History, n (%)*				0.062 ^b^			0.035 ^b^
No	2717 (82.2)	915 (82.7)	465 (78.9)		1102 (83.9)	235 (78.9)	
Yes	590 (17.8)	192 (17.3)	124 (21.1)		211 (16.1)	63 (21.1)	
*Family History Of Chronic Disease, n (%)*				0.211 ^b^			0.008 ^b^
No	685 (20.7)	247 (22.3)	116 (19.7)		279 (21.2)	43 (14.4)	
Yes	2622 (79.3)	860 (77.7)	473 (80.3)		1034 (78.8)	255 (85.6)	
*Smoking*, *n (%)*				0.006 ^b^			<0.001 ^b^
No	3244 (98.1)	1094 (98.8)	571 (96.9)		1295 (98.6)	284 (95.3)	
Yes	63 (1.9)	13 (1.2)	18 (3.1)		18 (1.4)	14 (4.7)	
*Alcohol*, *n (%)*				0.001 ^b^			0.459 ^b^
No	2514 (76.0)	887 (80.1)	432 (73.3)		979 (74.6)	216 (72.5)	
Yes	793 (24.0)	220 (19.9)	157 (26.7)		334 (25.4)	82 (27.5)	
*Regular Exercise, n (%)*				0.006 ^b^			<0.001 ^b^
No	1902 (57.5)	619 (55.9)	370 (62.8)		691 (52.6)	222 (74.5)	
Yes	1405 (42.5)	488 (44.1)	219 (37.2)		622 (47.4)	76 (25.5)	
*Cancer Screening, n (%)*				<0.001 ^b^			<0.001 ^b^
No	1374 (41.5)	399 (36.0)	290 (49.2)		525 (40.0)	160 (53.7)	
Yes	1933 (58.5)	708 (64.0)	299 (50.8)		788 (60.0)	138 (46.3)	

^a^ Mann–Whitney U Test, ^b^ Chi-Square Test, ^c^ Fisher’s exact test.

**Table 3:** Mixed effects logistic regression of factors associated with abnormal HADS in staff health screenings, 2018–2022.

Characteristic	*p*-Value	OR	95% CI	
			Lower	Upper
Age	0.001	0.967	0.948	0.987
*Year*	<0.001			
2018		1		
2019	0.724	1.059	0.77	1.456
2022	<0.001	2.284	1.644	3.174
Male (ref = Female)	0.286	1.413	0.749	2.666
*Race*	0.171			
Chinese		1		
Indian	0.973	1.012	0.514	1.993
Malay	0.402	1.262	0.732	2.174
Others	0.031	1.995	1.067	3.733
*Marital status*	0.750			
Married		1		
Single	0.619	1.118	0.720	1.735
Others	0.532	1.277	0.593	2.752
*Institution*	0.239			
Clinic A		1		
Clinic B	0.481	1.366	0.573	3.255
Clinic C	0.999	1.000	0.534	1.874
Clinic D	0.886	1.048	0.55	1.999
Clinic E	0.108	1.601	0.903	2.841
Clinic F	0.264	0.692	0.362	1.321
Clinic G	0.843	1.076	0.523	2.214
Headquarters	0.339	1.455	0.674	3.142
*Profession*	0.019			
Ancillary		1		
Administrative	0.260	0.693	0.365	1.312
Allied Health	0.301	0.694	0.347	1.387
Medical	0.004	0.391	0.207	0.739
Nursing	0.009	0.564	0.366	0.867
BMI	0.131	1.028	0.992	1.065
Hypertension	0.413	1.261	0.724	2.195
Hyperlipidemia	0.177	1.260	0.901	1.761
*Diabetes status*	0.284			
No diabetes		1		
Pre-Diabetes	0.141	0.483	0.183	1.273
Diabetes	0.428	0.728	0.333	1.595
Family history of chronic disease	0.104	1.384	0.935	2.049
Smoking	0.119	2.412	0.797	7.303
Alcohol user	0.027	1.558	1.053	2.307
Regular exercise	0.044	0.724	0.529	0.991
Long term medication use	0.407	1.309	0.693	2.475
Cancer screening	0.103	0.685	0.434	1.080
Significant past history	0.25	1.265	0.848	1.887

**Table 4:** Mixed effects logistic regression of factors associated with abnormal GHQ-12 in staff health screenings, 2023–2025.

Characteristic	*p*-Value	OR	95% CI	
			Lower	Upper
Age	0.002	0.955	0.928	0.983
*Year*	0.616			
2023		1		
2024	0.749	0.935	0.620	1.410
2025	0.334	0.802	0.513	1.255
Male (ref = Female)	0.865	0.934	0.426	2.050
*Race*	0.031			
Chinese		1		
Indian	0.779	0.875	0.344	2.224
Malay	0.05	1.903	1.001	3.620
Others	0.016	2.907	1.218	6.937
*Marital status*	0.521			
Married		1		
Single	0.514	1.225	0.665	2.255
Others	0.60	0.852	0.470	1.548
*Institution*	0.640			
Clinic A		1		
Clinic B	0.954	1.024	0.457	2.297
Clinic C	0.581	0.786	0.334	1.848
Clinic D	0.378	1.454	0.633	3.34
Clinic E	0.976	1.012	0.466	2.197
Clinic F	0.149	0.486	0.182	1.295
Clinic G	0.987	1.007	0.417	2.435
Headquarters	0.671	1.214	0.495	2.976
*Profession*	0.526			
Ancillary		1		
Administrative	0.69	1.179	0.526	2.641
Allied Health	0.405	0.652	0.238	1.783
Medical	0.194	0.532	0.205	1.380
Nursing	0.721	0.903	0.514	1.585
Others	0.225	0.373	0.076	1.833
BMI	0.736	0.993	0.952	1.035
Hypertension	0.662	1.191	0.544	2.608
Hyperlipidemia	0.988	1.003	0.654	1.539
*Diabetes status*	0.537			
No diabetes		1		
Pre-Diabetes	0.266	1.95	0.601	6.327
Diabetes	0.947	1.045	0.286	3.812
Family history of chronic disease	0.005	2.224	1.278	3.87
Smoking	0.004	5.786	1.753	19.099
Alcohol user	0.707	1.097	0.676	1.780
Regular exercise	<0.001	0.357	0.231	0.551
Long term medication use	0.744	0.847	0.311	2.303
Cancer screening	0.673	0.888	0.511	1.542
Significant past history	0.395	1.253	0.745	2.109

## Data Availability

The data presented in this study are available on request from the corresponding author.

## Supplementary Materials

The following supporting information can be downloaded at: https://www.mdpi.com/article/10.3390/healthcare14182947/s1, Table S1: Medical conditions considered as significant past medical history; Table S2: Linear mixed effects regression of factors associated with HADS scores in staff health screenings, 2018–2022; Table S3: Mixed effects logistic regression of factors associated with abnormal HADS scores in staff health screenings: subgroup analysis of frequent attenders, 2018–2022; Table S4: Linear mixed effects regression of factors associated with GHQ-12 scores in staff health screenings, 2023–2025; Table S5: Mixed effects logistic regression of factors associated with abnormal GHQ-12 scores in staff health screenings: subgroup analysis of frequent attenders, 2023–2025.
