# Supplementary material for: Psychological Distress Among Primary Care Professionals in Singapore: Repeated-Measures Analyses of Two Independent Observation Windows (HADS and GHQ-12)
Source: Healthcare (Basel). 2026 Sep 10;14(18):2947. doi: 10.3390/healthcare14182947 (PMC13606715; doi:10.3390/healthcare14182947)
Supplement: Supplementary file 1 [file healthcare-14-02947-s001.zip › healthcare-4496752-supplementary.pdf]

Supplementary Tables

Table S1. Medical conditions considered as significant past medical history.

Allergic Rhinitis

Amenorrhea

Anaemia

Ankylosing Spondylitis

Arrhythmia

Arotic valve stenosis

Asthma

Benign or malignant neoplasms

Chronic gastric issues

Dextrocardia

Eczema

Endometriosis

Epithelial basement membrane dystrophy

Fatty liver

Fibroid

Gestational diabetes

Glaucoma

Hysterectomy

Inflammatory bowel disease

Keratoconus

Kidney stone

Leukemia

Lupus

Mental health disorders

Migrane

Nasopharyngeal carcinoma

Neuropathy or spinal cord conditions

Obstructive sleep apnoea

Osteoarthritis

Osteoporosis

Polycystic ovarian syndrome

Prolapsed intervertebral disc

Rheumatoid arthritis

Scoliosis

Seizure

Sinusitis

Thalassemia

Thyroid disorders

Tuberculosis

Urticaria

Vasovagal Syncope

Table S2. Linear mixed effects regression of factors associated with HADS scores in staff health screenings, 2018–2022.

Characteristic	P-value	β	95% CI	
			Lower	Upper
**Age**	< 0.001	-0.07	-0.104	-0.036
**Year**	< 0.001			
2018		1		
2019	0.155	0.3	-0.114	0.714
2022	< 0.001	1.398	0.943	1.853
**Male** (ref = Female)	0.578	0.325	-0.821	1.471
**Race**	0.053			
Chinese		1		
Indian	0.328	0.584	-0.587	1.756
Malay	0.544	0.298	-0.665	1.262
Others	0.007	1.487	0.411	2.562
**Marital status**	0.089			
Married		1		
Single	0.831	0.08	-0.659	0.819
Others	0.028	1.382	0.149	2.614
**Institution**	0.797			
Clinic A		1		

Clinic B	0.995	-0.005	-1.482	1.473
Clinic C	0.736	0.188	-0.905	1.282
Clinic D	0.705	-0.217	-1.337	0.904
Clinic E	0.436	0.405	-0.616	1.426
Clinic F	0.393	-0.486	-1.602	0.63
Clinic G	0.624	0.315	-0.945	1.574
Headquarters	0.616	-0.325	-1.597	0.947
**Profession**	0.003			
Ancillary		1		
Administrative	0.523	0.313	-0.648	1.274
Allied Health	0.339	-0.512	-1.561	0.537
Medical	0.001	-1.776	-2.859	-0.694
Nursing	0.008	-1.016	-1.766	-0.266
**BMI**	0.075	0.057	-0.006	0.121
**Hypertension**	0.99	-0.005	-0.863	0.852
**Hyperlipidemia**	0.203	0.344	-0.185	0.873
**Diabetes**	0.66			
No diabetes		1		
Pre-Diabetes	0.365	-0.622	-1.97	0.725
Diabetes	0.743	-0.207	-1.443	1.029
**Family history of chronic disease**	0.167	0.429	-0.18	1.038
**Smoking**	0.034	1.927	0.148	3.706
**Alcohol user**	0.165	0.465	-0.192	1.121
**Regular exercise**	0.019	-0.565	-1.039	-0.091
**Long term medication use**	0.83	0.108	-0.881	1.097
**Cancer screening**	0.149	-0.559	-1.319	0.201
**Significant past history**	0.05	0.677	0.000	1.354

Table S3. Mixed effects logistic regression of factors associated with abnormal HADS scores in staff health screenings: subgroup analysis of frequent attenders, 2018–2022.

Characteristic	P-value	OR	95% CI	
			Lower	Upper
**Age**	0.01	0.959	0.928	0.99

**Year**	< 0.001			
2018		1		
2019	0.327	1.248	0.801	1.945
2022	< 0.001	2.816	1.807	4.39
**Male** (ref = Female)	0.949	0.966	0.34	2.746
**Race**	0.242			
Chinese		1		
Indian	0.739	0.839	0.299	2.356
Malay	0.243	1.632	0.718	3.71
Others	0.089	2.283	0.883	5.903
**Marital status**	0.364			
Married		1		
Single	0.46	0.77	0.385	1.539
Others	0.252	1.899	0.634	5.687
**Institution**	0.660			
Clinic A		1		
Clinic B	0.72	0.786	0.21	2.937
Clinic C	0.751	1.166	0.452	3.009
Clinic D	0.338	0.618	0.231	1.654
Clinic E	0.352	1.509	0.634	3.589
Clinic F	0.588	0.767	0.294	2.001
Clinic G	0.67	1.276	0.417	3.905
Headquarters	0.518	0.675	0.205	2.222
**Profession**	0.460			
Ancillary		1		
Administrative	0.661	1.26	0.448	3.544
Allied Health	0.837	1.11	0.41	3.01
Medical	0.942	1.038	0.38	2.835
Nursing	0.114	0.587	0.303	1.137
**BMI**	0.011	1.078	1.017	1.142
**Hypertension**	0.763	1.138	0.492	2.634
**Hyperlipidemia**	0.308	1.297	0.787	2.139
**Diabetes result**	0.976			

No diabetes		1		
Pre-Diabetes	0.825	0.847	0.193	3.719
Diabetes	0.996	0.997	0.249	3.989
**Family history of chronic disease**	0.182	1.485	0.831	2.655
**Smoking**	0.528	1.727	0.317	9.408
**Alcohol user**	0.444	1.257	0.699	2.261
**Regular exercise**	0.025	0.592	0.373	0.937
**Long term medication use**	0.828	1.107	0.441	2.776
**Cancer screening**	0.002	0.337	0.17	0.67
**Significant past history**	0.885	0.956	0.52	1.759

Table S4. Linear mixed effects regression of factors associated with GHQ-12 scores in staff health screenings, 2023–2025.

Characteristic	P-value	β	95% CI	
			Lower	Upper
**Age**	0.05	-0.012	-0.024	0.000
**Year**	0.231			
2023		1		
2024	0.17	-0.13	-0.315	0.055
2025	0.112	-0.159	-0.354	0.037
**Male** (ref = Female)	0.582	-0.102	-0.463	0.26
**Race**	0.219			
Chinese		1		
Indian	0.922	-0.02	-0.427	0.386
Malay	0.053	0.326	-0.005	0.656
Others	0.364	0.21	-0.244	0.664
**Marital status**	0.188			
Married		1		
Single	0.073	0.273	-0.026	0.572
Others	0.661	0.061	-0.211	0.332
**Institution**	0.484			
Clinic A		1		
Clinic B	0.924	-0.019	-0.407	0.369

Clinic C	0.54	-0.12	-0.504	0.264
Clinic D	0.351	0.194	-0.214	0.602
Clinic E	0.736	0.062	-0.296	0.419
Clinic F	0.123	-0.32	-0.726	0.087
Clinic G	0.908	0.024	-0.391	0.44
Headquarters	0.496	-0.149	-0.576	0.279
**Profession**	0.183			
Ancillary		1		
Administrative	0.331	0.197	-0.2	0.594
Allied Health	0.287	-0.222	-0.631	0.187
Medical	0.086	-0.348	-0.745	0.049
Nursing	0.33	-0.135	-0.407	0.137
Others	0.235	-0.321	-0.852	0.209
**BMI**	0.792	-0.003	-0.024	0.018
**Hypertension**	0.505	-0.118	-0.465	0.229
**Hyperlipidemia**	0.479	-0.071	-0.268	0.126
**Diabetes**	0.664			
No diabetes		1		
Pre-Diabetes	0.518	0.184	-0.373	0.741
Diabetes	0.471	0.207	-0.356	0.771
**Family history of chronic disease**	0.029	0.259	0.026	0.492
**Smoking**	< 0.001	2.194	1.501	2.888
**Alcohol user**	0.533	0.073	-0.156	0.301
**Regular exercise**	< 0.001	-0.406	-0.592	-0.22
**Long term medication use**	0.749	-0.064	-0.458	0.329
**Cancer screening**	0.55	-0.078	-0.335	0.178
**Significant past history**	0.06	0.243	-0.01	0.496

Table S5. Mixed effects logistic regression of factors associated with abnormal GHQ-12 scores in staff health screenings: subgroup analysis of frequent attenders, 2023–2025.

Characteristic	P-value	OR	95% CI	
			Lower	Upper
**Age**	0.109	0.972	0.939	1.006

**Year**	0.631			
2023		1		
2024	0.958	1.013	0.633	1.619
2025	0.406	0.807	0.488	1.337
**Male** (ref = Female)	0.487	1.454	0.506	4.178
**Race**	0.110			
Chinese		1		
Indian	0.819	0.873	0.273	2.789
Malay	0.125	1.869	0.84	4.156
Others	0.051	3.612	0.995	13.117
**Marital status**	0.537			
Married		1		
Single	0.27	1.502	0.73	3.093
Others	0.715	1.188	0.471	2.996
**Institution**	0.148			
Clinic A		1		
Clinic B	0.56	1.371	0.474	3.964
Clinic C	0.953	1.033	0.353	3.021
Clinic D	0.044	2.827	1.027	7.786
Clinic E	0.165	1.964	0.758	5.091
Clinic F	0.215	0.434	0.116	1.623
Clinic G	0.89	1.083	0.347	3.38
Headquarters	0.965	0.974	0.294	3.228
**Profession**	0.434			
Ancillary		1		
Administrative	0.207	1.964	0.688	5.61
Allied Health	0.778	0.843	0.258	2.756
Medical	0.269	0.492	0.14	1.731
Nursing	0.74	0.887	0.438	1.797
**BMI**	0.957	0.999	0.947	1.053
**Hypertension**	0.873	1.082	0.412	2.842
**Hyperlipidemia**	0.416	0.804	0.476	1.359
**Diabetes result**	0.796			

No diabetes		1		
Pre-Diabetes	0.501	1.627	0.395	6.698
Diabetes	0.955	1.042	0.254	4.269
**Family history of chronic disease**	0.096	1.751	0.905	3.386
**Smoking**	0.016	6.594	1.422	30.59
**Alcohol user**	0.878	1.048	0.579	1.895
**Regular exercise**	< 0.001	0.351	0.209	0.591
**Long term medication use**	0.646	0.763	0.24	2.427
**Cancer screening**	0.986	1.006	0.51	1.984
**Significant past history**	0.177	1.526	0.827	2.815
